# The AMADEUS score is not a sufficient predictor for functional outcome after autologous chondrocyte implantation (ACI) of the knee: data from the German Cartilage Registry (KnorpelRegister DGOU)

**DOI:** 10.1007/s00402-023-05037-z

**Published:** 2023-08-28

**Authors:** Tizian Heinz, Jan Oberfeld, Karsten Sebastian Luetkens, Philip Mark Anderson, Ioannis Stratos, Konstantin Horas, Thorsten Alexander Bley, Maximilian Rudert, Stephan Reppenhagen, Manuel Weißenberger

**Affiliations:** 1 1Department of Orthopaedic Surgery, University of Wuerzburg, Koenig-Ludwig-Haus, Brettreichstr. 11, 97074 Würzburg, Germany; 2 2Department of Diagnostic and Interventional Radiology, University Hospital Wuerzburg, Oberduerrbacherstr. 6, 97080 Würzburg, Germany

**Keywords:** Cartilage, AMADEUS, KOOS, IKDC, Knee, ACI

## Abstract

**Introduction:**

The AMADEUS (Area Measurement And DEpth and Underlying Structures) score has advanced to a commonly used tool for MRI-based chondral defect severity grading prior to cartilage knee surgery. It was the intention of this study to assess the AMADEUS for a potential correlation with clinical data by patient-reported outcome measures (PROMs).

**Methods:**

A total of 51 patients undergoing ACI (autologous chondrocyte implantation) between 2016 and 2022 were found eligible and retrospectively analyzed. All patients were registered in the German Cartilage Registry prior to surgery and follow-up data were collected using the Knee Osteoarthritis Outcome score (KOOS), the International Knee Documentation Committee (IKDC) Form and the numeric rating scale (NRS). Pre-operative MRI images were scored by three raters using the AMADEUS classification system, and an overall AMADEUS score was calculated which was subsequently correlated with pre- and post-operative PROMs.

**Results:**

Mean patient age was 32.67 ± 8.37 years and mean defect size area 343.04 mm^2^ ± 139.45 mm^2^. No correlative capacity of the pre- and postoperative IKDC, KOOS or NRS scores was found with the AMADEUS final score or any of its subscores. From the pre- to postoperative visit, a significant improvement of the PROMs (IKDC: 45.53 ± 21.00 vs. 59.83 ± 17.93, *p* = 0.04; KOOS Pain: 58.00 ± 16.70 vs. 76.06 ± 19.20, *p* = 0.03; KOOS ADL: 64.17 ± 18.76 vs. 82.11 ± 16.68, *p* < 0.01; KOOS Sports: 26.11 ± 18.52 vs. 50.56 ± 23.94, p = 0.01; KOOS QOL: 25.50 ± 14.26 ± 45.28 ± 19.03, *p* = 0.00) was found. Intraclass correlation coefficients showed an overall good interrater agreement for the AMADEUS total score (ICC = 0.75).

**Conclusions:**

Study results suggest no correlative capacity of the AMADEUS with routinely used PROMs in patients undergoing ACI. Therefore, radiographically assessed cartilage defect characteristics poorly translate to pre- and postoperative patient-reported outcome data.

## Introduction

Cartilage defects of the knee remain a challenging pathology in the fields of orthopedics and sports medicine. It is estimated that cartilage defects are found in approximately 12% of the overall population, increasing to a total of 63% in patients with records of traumatic knee joint injury [1, 2]. As hyaline cartilage of diarthrodial joints is unique in many aspects, like being a aneuronal, avascular and alymphatic tissue allowing for extremely low-frictional movements, its regeneration potential is likewise complex, resulting in limited ability for self-repair [3]. At the same time, cartilage defects have a high potential for considerable pain and disability with impact on patient mobility and quality of life. Moreover, cartilage defects predispose to early degenerative joint deterioration resulting in early onset osteoarthritis [2, 4]. Therefore, reliable treatment options for chondral defects are of upmost importance both for restoring pain-free mobility and preventing long-term sequelae like early onset osteoarthritis.

Autologous chondrocyte implantation (ACI), since its first description by Matts Brittberg in 1994 [5], has evolved to a first line treatment modality for large (> 2.5 cm^2^) full-thickness chondral defects in the non-osteoarthritic patient with satisfactory results in the long-term period (10–20 years) [6–9]. While MRI-based scoring instruments for the postoperative evaluation of cartilage repair tissue are commonly employed, like the MOCART (magnetic resonance observation of cartilage RT) score, MRI scoring instruments for the evaluation of the chondral defects at the preoperative visit are sparse. Recently, Jungmann et al. reported on a novel MRI scoring instrument for detailed preoperative chondral defect severity grading (AMADEUS—Area Measurement And DEpth and Underlying Structures) [10]. By evaluation of four distinctive parameters of the cartilage defect (1—defect size, 2—defect depth, 3—integrity of subchondral bone, 4—presence of surrounding bone marrow edema), a score resulting from 0 to 100 is formed. While correlative association of radiographic data with clinical data in terms of patient-reported outcome measures (PROMs) are well investigated for the cartilage repair tissue [11, 12], correlative data of the preoperatively encountered focal cartilage defect with clinical outcome scores are lacking. Therefore, the primary aim of this study was to evaluate the correlative association of the recently developed MRI-based AMADEUS score with clinical outcome scores pre- and postoperatively in patients undergoing ACI for full-thickness chondral defects of the knee joint. Based on the present literature trying to connect postoperative MRI data to functional outcome scores [13, 14], only a weak or no significant correlation was hypothesized to be found between the preoperative AMADEUS score and clinical outcome data.

## Materials and methods

### Study population and design

This retrospective study encompassed a total of 51 patients undergoing ACI for full chondral cartilage defects of the knee during April 2016 to October 2022 at a single orthopedic university center. Patients were asked to register in the nationwide German Articular Cartilage Registry of the German Association for Orthopedics and Traumatology (DGOU—“Deutsche Gesellschaft für Orthopädie und Unfallchirurgie”) prior to planned surgery. Participation was voluntary and did not influence the surgical procedure and postoperative rehabilitation protocol. Eligibility criteria for this study were defined as follows: (1) a full-thickness chondral defect of the knee diagnosed by MRI and arthroscopy, (2) conducted ACI procedure as indicated by a senior orthopedic consultant, (3) consent of the patient for participation at the Cartilage Registry, (4) Minimum age of 18 years at the time of participation. Inflammatory arthritis or previous ligamentous injury were defined as exclusion criteria for this study.

All patients were evaluated retrospectively by means of achieved medical records and monocentric data from the German Cartilage Registry. Only monocentric patients were eligible for evaluation due to a lack of radiographic data transferability.

The German Cartilage Registry (“KnorpelRegister DGOU”) was conducted in accordance with the Declaration of Helsinki of 1964 and registered at germanctr.de (DRKS00005617). Primary ethical approval was given by the ethics committee at the University of Freiburg (No. 520/14). In addition, the registration of data was approved by the local ethics committee at the University of Wuerzburg (No. 333/15).

### German Articular Cartilage Registry

The German Cartilage Registry is a nationwide registry of multicentric structure for patients intended to undergo cartilage repair surgery of the knee joint. The registry is fully digitalized and patient and surgery-related information is entered securely online after logging into a password secured user area. Approximately, 140 orthopedic centers in Germany, Austria and Switzerland are contributing data to the German Cartilage Registry. Patient-specific characteristics such as age, sex, weight, body mass index (BMI) as well as intraoperative defect specific parameters (e.g. defect size, defect localization, ICRS-grade of the defect, operative procedure applied) are entered by the physician. Meanwhile, the patient is asked to fill in validated questionnaires such as the Knee Osteoarthritis Outcome Score (KOOS), International Knee Documentation Committee (IKDC) and the numeric rating scale (NRS) for pain to assess clinical symptoms and functional outcome. Links to the questionnaires are sent out automatically to the patients’ email-address at specific time points and are accessible online through the web-browser for 4 weeks after the link was sent out (preoperatively as well as 6, 12, 24, 36, 60 and 120 months after intervention).

### MR image assessment and AMADEUS grading

**Fig. 1:**
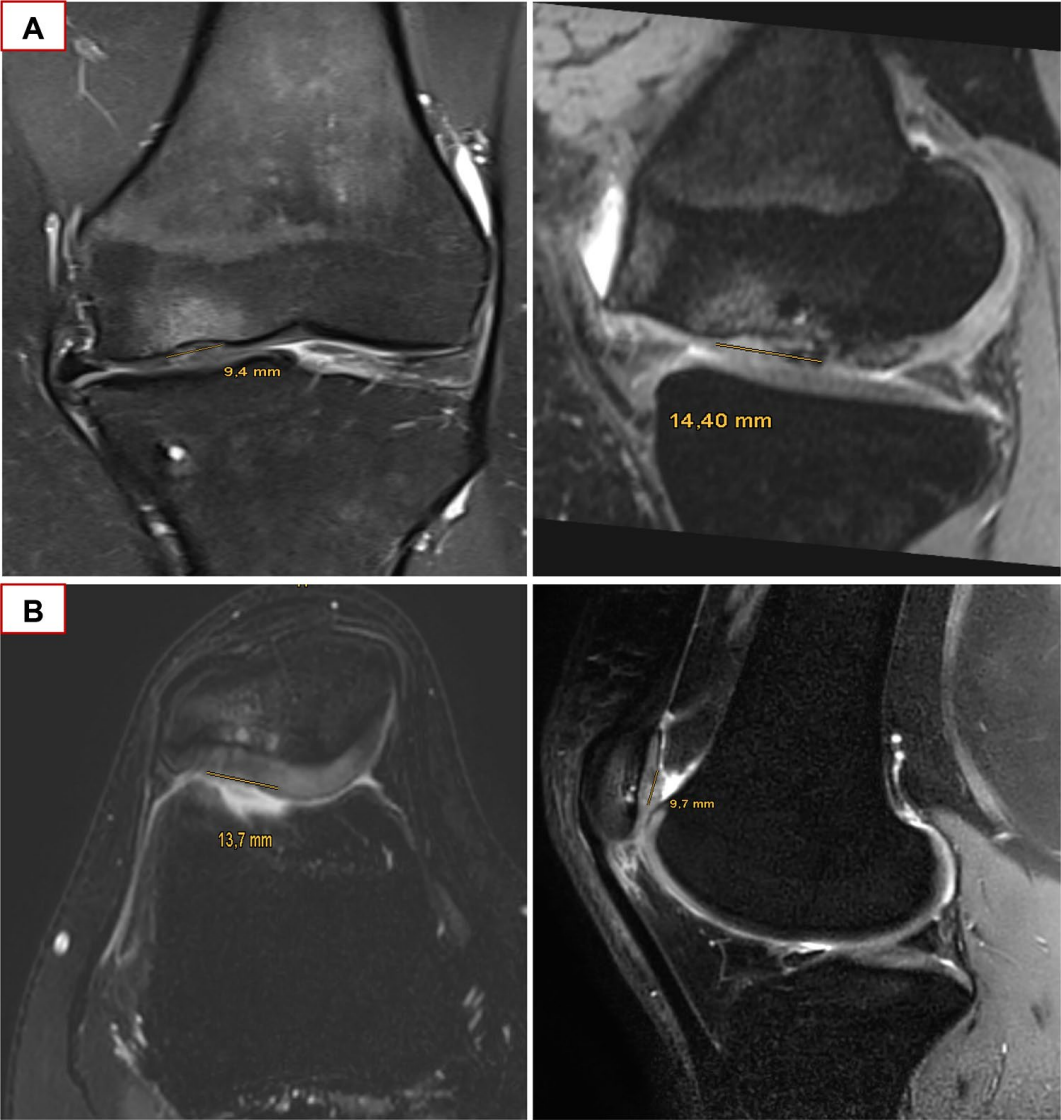
Illustration of MRI-based AMADEUS score grading. Exemplary illustration of MRI image evaluation according to the AMADEUS scoring protocol. **A** Proton density weighted turbo spin echo fat saturated (PD-TSE-FS, coronal and sagittal view) MRI images revealing a full-thickness chondral defect of the medial femoral condyle. The defect area was measured as 1.35 cm^2^. A small bony defect underneath the cartilage defect area is clearly visible. A Bone marrow edema is surrounding the cartilage defect area. This corresponds to a total AMADEUS score of 50 points: First digit “defect area” = 30 points (> 1 cm^2^ to ≤ 2 cm^2^); Second digit “defect depth” = 0 points (full-thickness chondral defect); third digit “underlying structure” = 20 points (bony defect < 5 mm); fourth digit “bone marrow edema” = 0 points (presence of bone marrow edema). **B** Proton density weighted turbo spin echo spectral presaturation with inversion recovery (PD-TSE-SPIR, axial and coronal view) MRI images demonstrating a full-thickness chondral defect of the patellar surface. The defect area was measured as 1.33 cm^2^. This corresponds to a total AMADEUS score of 70 points: First digit “defect area” = 30 points (< 1 cm^2^ to ≤ 2 cm^2^); second digit “defect depth” = 0 points (full-thickness chondral defect); third digit “underlying structure” = 30 points (no bony defect); fourth digit = 10 points (no bone marrow edema)

Intermediate-weighted (IM), T2-weighted fast spin echo (FSE) or proton-density (PD) weighted MR images in combination with T2-weighted FSE images in at least two planes were found suitable for the AMADEUS scoring. Furthermore, for evaluation of bone marrow edemas a T1-weighted image was additionally required. Three different raters had independently scored the MR images at the preoperative visit: One radiologist trained in musculoskeletal imaging and two residents in orthopedics and traumatology. AMADEUS scoring was performed according to the scoring instruction described by Jungmann et al. [10]. Briefly, the cartilage defect area was measured by multiplying the sagittal and coronal (for condyle and tibial defects) or sagittal and transverse (for retropatellar defects) defect diameters. Defect depth was evaluated incrementally as no defect, signal alterations, partial-thickness defects and full-thickness defects. Moreover, the underlying structure of the defect was scored as intact subchondral lamina, small (< 5 mm) and extensive (≥ 5 mm) subchondral bony defects. In addition, the presence of bone marrow edemas surrounding the cartilage defect area was evaluated. Based on the four AMADEUS subscore items a total AMADEUS score and AMADEUS grade was formed (Fig. 1). All MRI images were digitally stored and are accessible via Picture Archiving Communication System (PACS).

### Patient-reported outcome measures (PROMs)

Upon admission to surgery patients were voluntarily registered at the German Cartilage Registry and patient-specific questionnaires were send out to the patients asking for symptoms, knee function, pain and sports activity prior to surgery. The questionnaires evaluating the preoperative condition were completed by the patients either before surgery or within a week after surgery. Specifically, the IKDC, KOOS and NRS were evaluated. The clinical important difference (CID) and patient acceptable symptomatic state (PASS) for the IKDC has been reported to be as high as 9.2 (CID) and 62.1 for patient undergoing cartilage repair procedures of the knee joint. (PASS) [15]. PASS and CID scores for the KOOS subscales range from 43.8 to 72.2 and 8.8–30.0, respectively [15]. Furthermore, questionnaires were sent out automatically six and twelve months after surgery for evaluation of the postoperative condition. All questionnaires were filled out online via a weblink sent to the patient.

The IKDC 2000 Subjective Knee Evaluation Form was drafted by a panel of leading knee surgeons and finally released in 1998 encompassing a set of 18 items in three categories [16]. The IKDC was designed to evaluate daily knee function, symptoms and sports activity in patients with a variety of knee pathologies. Meanwhile, the IKDC has been validated for patients undergoing cartilage repair surgery [17].

The KOOS was evaluated by scoring its five subscores (Pain, Symptoms, Function, ADL, QOL) separately, according to established scoring guidelines [18]. Consistency and reliability of the KOOS have been validated for patients after cartilage repair surgery [19].

### Surgical technique

A standard two-stage ACI procedure was used as described elsewhere [20, 21]. First, a diagnostic arthroscopy was performed and the cartilage defect area was evaluated. During routine arthroscopy, one to two small full-thickness cartilage cylinders of one to two mm in diameter were harvested from non-weight bearing areas around the intercondylar notch. Subsequently, the harvested cells were sent to a specialized laboratory for cell expansion, chondrogenic differentiation and seeding in a collagen matrix (Novocart 3D, TeTeC, Reutlingen, Germany). Three weeks following the cell harvesting, the cell loaded matrix was reimplanted filling the cartilage defect area via a mini open knee arthrotomy. A standardized postoperative rehabilitation program was applied afterwards, encompassing a limited weight-bearing for at least 6 weeks accompanied by continuous passive motion (CPM) therapy.

## Statistics

Statistical analysis was conducted with the aid of a statistical software package (SPSS version 27, IBM Corp.) and a *p* value of 0.05 was set as level of significance. Descriptive data is presented as mean values with standard deviation (SD) or relative numbers (percentage %). Normal distribution was tested using the Kolmogorov–Smirnov and Shapiro–Wilk test. In case of not-normally distributed data, non-parametric testing was used. PROMs were analyzed by mean values and SD. Mean values of the PROMs were compared using a one-way ANOVA analysis for group differences at a single timepoint or a dependent *t* test for differences in the mean values over time. Associations between radiographic data and clinical data (PROMs) were assessed by a correlation analysis using the non-parametric Spearman rank correlation coefficient (SCC). Interobserver agreement for radiographic data was analyzed by calculating the intraclass correlation coefficients (ICC) based on a mean rating (*k* = 3), consistency, two-way random-effects model. An ICC of less than 0.5, 0.5–0.75, 0.75–0.9 and above 0.9 were interpreted as poor, moderate, good and excellent reliability based on current literature of Koo et al. [22]. An a priori sample size calculation was performed aiming for a statistical power of 0.80 and an alpha level of 0.05. Therefore, the correlation coefficient for the null hypothesis (*H*_0_: No correlative association between the AMADEUS and PROM Data exist) was set at 0.00. For the alternative hypothesis (*H*_1_: Moderate to strong correlative association between AMADEUS and PROM Data), a correlation coefficient of at least 0.4 was set and a sample size of at least 37 patients was calculated.

## Results

A total of 51 patients (33 males and 18 females) adding up to 53 cartilage defects were included in the study. Most defects were located at the medial femoral condyle (41.2%) followed by the lateral femoral condyle (19.6%) and the retropatellar surface (14.5%). Detailed patient and cartilage defect characteristics are displayed in Table 1.

**Table 1:** Overview of the patient and cartilage defect characteristics

Descriptive item	Mean (± SD) or total/relative number (percentage %)
Gender	
Male	33 (64.7%)
Female	18 (35.3%)
Age (years)	32.67 ± 8.37
BMI	27.33 ± 5.61 (kg/m^2^)
Number of defects	53
Defect size	343.04 mm^2^ ± 139.45 mm^2^
Defect localization	
Medial femoral condyle	21 (41.2%)
Lateral femoral condyle	10 (19.6%)
Trochlea	3 (5.9%)
Patella	27 (14.5%)
Tibial plateau	2 (3.9%)
Smoking	
Yes	10 (19.6%)
No	41 (80.4%)

**Fig. 2:**
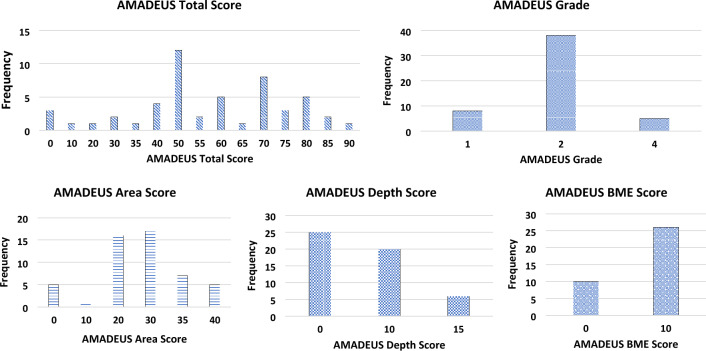
Distributional patterns of the AMADEUS subscores within the patient collective

The mean AMADEUS total score was calculated as 55.0 ± 22.16 points. The distribution patterns of the AMADEUS total score with all its four subscores (area defect score, depth score, underlying structure score, bone marrow edema score) are presented in Fig. 2. As perceivable from the subscore distribution patterns, full-thickness cartilage defects were most common in the patient collective (48.1% of cartilage defects with an AMADEUS depth score of zero points, corresponding to a full-thickness cartilage defect). However, despite the full-thickness defect structure, no or only small bony defects were present in most of the cases (corresponding to 71% of cartilage defects with an underlying structure score of ≥ 20 points).

Regarding the PROMs, mean values of the IKDC and of the five subscales of the KOOS are presented in Table 2. Four of five KOOS subscale items improved significantly from the preoperative visit to the postoperative visit 12 months following the cartilage repair procedure. The mean IKDC score similarly showed a statistically significant improvement from the pre- to the postoperative visit (Table 2). Mean KOOS subscale scores as well as the mean IKDC and NRS score did not differ significantly with regard to the defect severity grading according to the AMADEUS grading scale (Table 3) or with respect to the defect location.

**Table 2:** Overview of the patient-reported outcomes scores at the pre- and postoperative visit

	KOOS symptoms	KOOS pain	KOOS ADL	KOOS sports	KOOS QOL	IKDC	NRS
Preoperative	50.28 ± 13.88	58.00 ± 16.70	64.17 ± 18.76	26.11 ± 18.52	25.50 ± 14.26	45.53 ± 21.00	3.25 ± 2.57
6 months	49.94 ± 11.03	66.48 ± 18.77	73.52 ± 18.93	34.00 ± 26.92	39.65 ± 21.46	48.34 ± 15.32	3.25 ± 2.00
12 months	54.11 ± 11.45	76.06 ± 19.20	82.11 ± 16.68	50.56 ± 23.94	45.28 ± 19.03	59.83 ± 17.93	2.63 ± 2.22
*p* value (preoperative vs. 6 month)	0.68	0.68	0.56	0.86	**0.04**	0.37	0.99
*p* value (preoperative vs. 12 months)	0.45	**0.01**	**0.00**	**0.01**	**0.00**	**0.04**	0.48
*p* value (6 months vs. 12 months)	0.28	**0.03**	**0.04**	0.12	0.20	**0.01**	0.62

The upper half of the table depicts the mean IKDC, KOOS and NRS scores of the patient collective. In the lower half of the table, *p* values of a dependent *t* test of the respective PROM are presented

Bold values indicate significance (*p* < 0.05)

**Table 3:** Mean pre- and postoperative IKDC scores displayed with respect to the incremental AMADEUS defect severity grading

AMADEUS grade	IKDC preoperative	IKDC 6 months	IKDC 12 months
1	45.83 ± 26.06	42.07 ± 17.59	53.64 ± 11.57
2	44.83 ± 20.03	49.56 ± 15.44	65.51 ± 15.62
4	55.17 ± 12.27	49.04 ± 12.61	43.29 ± 24.39
*p* value	0.58	0.62	0.13

The last row indicates the *p* values for mean value differences between the AMADEUS gradings at a specific time point

Correlation analysis revealed no statistically significant correlative association between the PROMs (IKDC, NRS and all five KOOS subscale scores) and the AMADEUS total score at the preoperative and postoperative (six months and twelve months) visit. The same finding applied to the AMADEUS subscale scores with the evaluated PROMs (Tables 4 and 5).

**Table 4:** Correlation analysis of the AMADEUS and its subscores with the pre- and postoperatively obtained IKDC scores

	AMADEUS grade		AMADEUS total score		Defect size score		Area score		Defect depth score		Underlying structure score		BME score	
	*R*	*p*	*R*	*p*	*R*	*p*	*R*	*p*	*R*	*p*	*R*	*p*	*R*	*p*
IKDC preoperative	0.15	0.32	− 0.04	0.77	0.02	0.89	− 0.03	0.83	− 0.07	0.63	− 0.04	0.79	− 0.03	0.83
IKDC 6 months	0.12	0.52	− 0.25	0.18	− 0.11	0.56	− 0.01	0.95	− 0.07	0.69	− 0.21	0.26	− 0.17	0.36
IKDC 12 months	− 0.09	0.71	− 0.00	0.99	0.07	0.79	− 0.19	0.46	0.05	0.85	0.07	0.79	0.08	0.77

*R* Spearman rho

**Table 5:** Correlation analysis of the AMADEUS and its subscores with the pre- and postoperatively obtained KOOS scores

	AMADEUS grade		AMADEUS total score		Defect size score		Area score		Defect depth score		Underlying structure score		BME score	
	*R*	*p*	*R*	*p*	*R*	*p*	*R*	*p*	*R*	*p*	*R*	*p*	*R*	*p*
KOOS pain	− 0.05	0.74	0.03	0.82	− 0.08	0.60	0.03	0.82	0.01	0.94	0.06	0.66	− 0.20	0.15
KOOS symptoms	0.07	0.64	− 0.15	0.31	− 0.01	0.95	− 0.02	0.88	− 0.32	0.02	− 0.08	0.58	− 0.01	0.94
KOOS ADL	− 0.03	0.84	− 0.08	0.56	0.03	0.86	− 0.03	0.84	− 0.08	0.57	− 0.00	0.97	− 0.09	0.52
KOOS QOL	0.21	0.13	− 0.15	0.31	− 0.04	0.81	− 0.00	0.99	− 0.05	0.72	− 0.14	0.33	− 0.20	0.16
KOOS sports	0.09	0.53	− 0.10	0.49	− 0.05	0.72	0.02	0.89	− 0.00	0.98	− 0.12	0.42	− 0.11	0.43

*R* Spearman rho

Intraclass correlation coefficients showed an overall good interrater agreement for the AMADEUS total score (ICC = 0.75) (Table 6). The AMADEUS subscore items showed a moderate interrater reliability, with only a poor interrater agreement for the AMADEUS depth score (ICC = 0.30).

**Table 6:** Intraclass correlation coefficients for interobserver reliability of the AMADEUS and its subscores

Item	ICC (95% CI)
AMADEUS total score	0.75 (0.59 to 0.85)
AMADEUS grade	0.39 (0.03 to 0.63)
AMADEUS area score	0.53 (0.24 to 0.72)
AMADEUS depth score	0.30 (− 0.12 to 0.58)
AMADEUS BME score	0.69 (0.50 to 0.81)
AMADEUS underlying score	0.69 (0.50 to 0.81)

## Discussion

Imaging modalities such as MRI have long been used primarily as a diagnostic tool in the fields of cartilage surgery. However, with the increasing ability to accurately assess cartilage defect areas due to enhanced MRI sequences and scanning techniques [23–25], a growing interest for a potential connection of MRI data with clinical outcome data has evolved. Presumably, the linkage of MRI-based cartilage defect grading with clinical outcome data would be beneficial for a profound and streamlined assignment of patients to the most appropriate treatment options, thus improving overall clinical outcome. Moreover, a correlative capacity of the imaging data with the pre- and postoperative clinical outcome would improve outcome predictability of the planned surgical procedure, thus easing patient communication regarding their prospective rehabilitation potential. So far, profound correlative data of the AMADEUS with both pre- and postoperative clinical outcome measurements in patients undergoing ACI are lacking.

As a main result of this study no correlative association was found between the AMADEUS total score or any of its subscores with clinical outcome data (IKDC, KOOS and NRS) both pre- and postoperatively. While some previous studies have tried to connect the preoperative AMADEUS score with postoperative PROM data, this is the first study evaluating specifically the IKDC and KOOS score for a potential correlation with the preoperative imaging data. Massen et al. analyzed 27 patients undergoing a single-step minced cartilage procedure for full-thickness chondral defects and were unable to find a correlative relationship between the AMADEUS score and clinical data by means of the numeric rating scale [26]. Similarly, Jung et al. could not demonstrate a relationship of the AMADEUS and Lysholm score in patients undergoing matrix-associated ACI with autologous bone grafting [27]. So far, only a weak correlation of the AMADEUS with the preoperatively evaluated Core Outcome Measures Index (COMI) score has been reported [28]. Consequently, cartilage lesion characteristics poorly translate to clinical data in terms of knee pain and function. While this finding is well recognized and extensively discussed in literature for the preoperative setting [29, 30], the present study suggests that cartilage lesion characteristics do also not influence the postoperative outcome measures in patients undergoing ACI. Furthermore, the location of the cartilage defect within the knee joint did not impact the patient reported pain or function. Interestingly, the presence or absence of a bone marrow edema surrounding the cartilage defect area did not influence pre- or postoperative patient reported pain or function, yet some authors were able to demonstrate a correlative relationship between the presence of bone marrow edema and clinical outcome data [31, 32]. Specifically, in patients undergoing high tibial valgus osteotomy for medial compartment chondral defects, the presence of bone marrow edema has been shown to be positively correlated with the KOOS symptoms subscale score at the preoperative visit [33]. This suggests that the correlative capacity of the AMADEUS may also be dependent on the cartilage repair procedure applied. The limited compliance of radiological data with clinical outcome data is thereby not an entirely new finding, as it has already been demonstrated with the MOCART score for postoperative MRI-based cartilage repair tissue evaluation [11, 34]. The reasons for this limited correlative capacity may be of a multilevel nature: The large number of variables of compound scores like the AMADEUS or the MOCART may negatively influence a potential correlative association [28, 35]. Moreover, there may be several other variables that are not captured by radiographic measurement but may greatly influence patient-reported outcomes, like patient expectations and communication [36, 37]. Patient-specific factors such as duration of symptoms, comorbidities, BMI and mental health status are typically not captured by radiographic data but may also have a significant impact on patient-reported outcome parameters [28].

While several attempts have been made to test for a correlation of the AMADEUS with PROMs in patients undergoing ACI, the present study adds some essential core insights to this subject: Firstly, this is the first study adding the KOOS score for a potential correlative association with the AMADEUS. This is of particular importance, as the KOOS yields a frequently used patient-reported measurement specifically validated for patients undergoing cartilage repair surgery [19]. Notably, out of eleven PROMs both the IKDC and KOOS represent the questionnaires most important and useful to patients with knee related problems [38]. However, the comparison of objective radiographic data with subjective PROM still warrants a potential for bias by data inconsistency. Secondly, the preoperatively obtained AMADEUS has not been studied for a correlative impact with PROMs at the postoperative visit. However, it is important to point out that the relatively small number of patients could be a limiting factor on the study results and a lager sample size might have added additional strength to the study design.

## Conclusion

The AMADEUS score is a valuable tool for accurate and detailed grading and description of chondral defects at the preoperative stage. However, it is not able to predict the clinical outcome of patients undergoing ACI for cartilage defect repair. Therefore, the AMADEUS score should be considered a purely radiographic score with very limited applicability to the clinical setting.

## Data Availability

The data that support the findings of this study are available from the corresponding author, MW, upon reasonable request.
